# Multiple primary brain abscesses in a 14 years old immunocompetent boy: A tale of Proteus mirabilis infestation from Pakistan

**DOI:** 10.12669/pjms.40.12(PINS).11033

**Published:** 2024-12

**Authors:** Tariq Imran Khokhar, Haseeb Mehmood Qadri, Ibreeza Fatima, Abdul Ghafoor, Muhammad Talha Bilal

**Affiliations:** 1 Tariq Imran Khokhar, Associate Professor, Department of Neurosurgery, Unit-I, Punjab Institute of Neurosciences, Lahore, Pakistan; 2 Haseeb Mehmood Qadri, Post Graduate Resident, Department of Neurosurgery, Unit-I, Punjab Institute of Neurosciences, Lahore, Pakistan; 3 Ibreeza Fatima, Post Graduate Resident, Department of Neurosurgery, Unit-I, Punjab Institute of Neurosciences, Lahore, Pakistan; 4 Abdul Ghafoor, Senior Registrar, Department of Neurosurgery, Unit-I, Punjab Institute of Neurosciences, Lahore, Pakistan; 5 Muhammad Talha Bilal, Post Graduate Resident, Department of Neurosurgery, Unit-I, Punjab Institute of Neurosciences, Lahore, Pakistan

**Keywords:** Brain abscess, Gram negative bacteria, Multiple brain abscesses, Pakistan, Proteus mirabilis

## Abstract

Brain abscess (BA) formation that may be due to due to Gram-positive bacteria commonly and less commonly due Gram-negative bacteria affects pediatric population. Most cases are secondary to the involvement of ear, nose, throat and sinuses (ENTS). We describe a rare case of a 14 year old patient presenting with generalized tonic clonic seizures for the last six months associated with fever, vomiting and headache. ENTS examination was normal. Neuroimaging was suggestive of multiple left fronto-pariettal space occupying lesions. Modified pterional craniotomy was done to excise the multiloculated, encapsulated lesion with yellow-green pus. Culture sensitivity yielded *Proteus mirabilis*. Patient was discharged from the hospital uneventfully. The diagnosis of multiple primary brain abscesses secondary to *Proteus mirabilis* is very rare and it should be considered in the list of differentials of multiple brain abscesses when the systematic examination is normal. Early surgical excision is the key to a successful outcome.

## INTRODUCTION

Brain abscess (BA) is defined as a localized collection of purulent content with cellular debris within brain parenchyma. The existing scientific literature documents an incidence of one to two percent in the West and up to eight percent in third-world countries.^1^ The occurrence of BAs is more commonly seen in adults than pediatric population and approximately twenty-five percent of all cases affect pediatric population.^2^,^3^ Sinusitis, otitis and dental infections are frequently noted causes of brain abscess owing to the local spread of infection. Secondary BAs are more common than primary cases.^1^ Risk factors can be acute, such as untreated otorhinolayrngological infections or head trauma and chronic, like uncorrected congenital heart defects or immunosuppression.^3^ Rupture of BA into the ventricular cavity or herniation of brain secondary to the mass effect caused by BAs is associated with fatal outcomes.^1^,^2^

Staphylococci and Streptococci are the most common bacterial causes of pediatric brain abscesses (PBAs) worldwide and a similar pattern is noted in Pakistan.^2^,^3^,^4^ Gram-negative organisms are the uncommon causative agents in PBAs. There is extremely limited scientific literature documenting Proteus species as the cause of BA in neonates. However, no such study has been documented in Pakistan in neonates or children. To the best of our comprehensive literature search using PubMed and Google Scholar, this shall be the first case of primary BA due to *Proteus mirabilis* in a 14 years old immunocompetent boy from Pakistan.

## CASE PRESENTATION

A 14 years old boy presented to neurosurgical outpatient clinic in January 2024 with the presenting complaints of generalized tonic clonic seizures for the last six months associated with high-grade fever, vomiting and headache off-and-on. Medical history was significant of two episodes of seizures immediately after birth, that subsided with anti-epileptics for two months and there was no recurrence of seizures afterwards. On clinical examination, he was well oriented and conscious. Neurological examination of sensory and motor system was normal.

**Fig.1:**
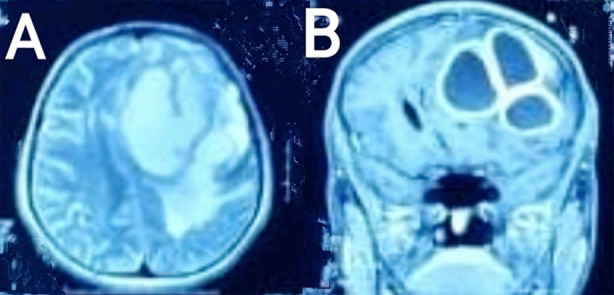
Contrast-enhanced magnetic resonance imaging (MRI) brain. A. Axial view, T2-weighted image showing left fronto-parietal multiple hyperintense space occupying lesions causing midline shift and B. Coronal view, T1-weighted image showing multiple hypointense lesions with ring enhancement.

On laboratory investigations, neutrophilic leukocytosis of 14,000 cells/µL with an erythrocyte sedimentation rate (ESR) of 34 mm/hour was found. Non-contrast enhanced computerized tomography (NCE-CT) of brain showed left fronto-parietal space occupying lesion (SOL) with calcifications. Contrast-enhanced magnetic resonance imaging (CE-MRI) brain was suggestive of left fronto-parietal SOL having mass effect and midline shift, appearing hypointense on T1-weighted image and hyperintense on T2-weighted image (Fig.1A and 1B). Sequence of T2-fluid attenuated inversion recovery (FLAIR) showed peripheral post-contrast enhancement and predominantly central diffusion restriction on diffusion-weighted image / apparent diffusion coefficient (DWI/ADC) images. Brain abscess secondary to Toxoplasma, Rubella, Cytomegalovirus, Herpes simplex (TORCH) infections and metastatic deposits were the main differential diagnoses. Investigations were ordered to rule out systematic sources of inflammatory and neoplastic pathologies. CT abdomen, pelvis and thorax were not suggestive of primary SOL. Electrocardiography and echocardiography were normal.

**Fig.2:**
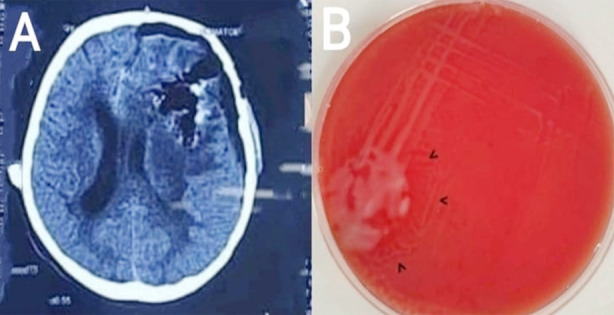
**A)** Non-contrast computerized tomography (CT) brain showing post-operative changes with no new pathology. **B)** Swarming motility of Proteus mirabilis on blood agar marked with black arrowheads.

Left fronto-parietal modified pterional craniotomy and excision of abscess was done. Multi-loculated, yellowish-green pus of 55 ml was aspirated initially. The calcifications were addressed and complete excision of the abscess with capsule was achieved. There were no immediate post-operative complications and the patient was recovered from anesthesia smoothly. Empirically, he was given intravenous (IV) vancomycin 750mg TDS and ceftriaxone 1gram BD for a week during in-patient stay. Meanwhile, the histopathology and microbiology report revealed dense acute and chronic inflammation, secondary to abscess formation by Proteus mirabilis (Fig.2A). As his clinical examination and laboratory tests were normal for all other systems, the diagnosis of primary, multiple brain abscesses by Proteus mirabilis was made. Based on the report of culture sensitivity, he was advised oral formulation of cefixime once daily for a week and single anti-epileptic for a month. However, the patient returned after three weeks with an episode of generalized tonic clonic seizure. NCE-CT brain was ordered immediately and it was normal (Fig.2B). Contrast-enhanced CT brain (CE-CT) was later planned which was also normal with post-operative changes. We suspected disruption in drug dosing or intake. He was prescribed double anti-epileptics for a month. The patient is living a healthy life and his anti-epileptic regimen is being tapered.

### Consent for publication:

Consent was obtained from the patient for the publication of this case report and the accompanying images.

## DISCUSSION

Cumulatively, otogenic, sinogenic, cardiogenic and odontogenic foci have been implicated as the cause of pediatric brain abscess (PBA) in 70%-90% cases according to a recent review by Mameli et al.^3^ Primary cases of PBA are not well-studied and documented uncommonly. We made the diagnosis of PBA in our patient after ruling out all other possible diagnosis and primary foci with complete physical examination and a series of laboratory and radiological investigations.

The identification of Proteus mirabilis PBA in a neonate date back to the year 1978 when Darby et al. reported it in a newborn of at his fourth day of life.^5^ Gram-negative bacteria are the usual cause of neonatal BA, mainly the species of Citrobacter, Proteus and less commonly Eschirechia and Serratia.^3^ However, the patient in our case is a 14 years old school-going boy with primary BA with Proteus mirabilis is a rare occurrence at this age.

Kanu and colleagues report the classic triad of fever, headache and focal neurologic deficit in 35% children in their retrospective study conducted in Nigeria over a period of 11 years.^4^ But this classic triad was not present in our patient, who had fever, headache and vomiting for the last six months. The long duration of presentation in our case could be attributed to the lack of awareness and resources in patients with rural background.

Recent literature defines a variety of common locations of PBAs. A prospective Pakistani study documented frontal lobe as the most common site of PBA in 36% patients,^2^ while an Indian study by Prasad et al. highlighted temporal lobe as the most frequently affected lobe in 36. 7% cases with history of chronic ear discharge in half of the patients.^1^,^2^ The involvement of two lobes i.e., frontal and parietal lobe in our patient is backed by the finding of multiple sites of BA in this case. The extensive involvement could also be due to the prolonged duration of symptoms and hence complexity of the disease. Kanu et al. found a striking left-laterality of multiple infratentorial BAs in 40% patients. This is in agreement with the left-sided presentation of multiple abscesses in our patient.^4^

Magnetic resonance imaging (MRI) is considered as the radiological investigation of choice to diagnose BA due to its more enhanced resolution and lower risk of toxicity by contrast compared to computerized tomography (CT). The usual findings of hyperintensity on T1-weighted image and hypointensity on T2-weighted image for a pyogenic BA were not found in the patient under study. Hyperintensity on T2-weighted image is well-explained by thick contents of BA in the current case, which in turn is attributed to high protein component significant of chronic abscesses.^3^

Third-generation cephalosporins have been used by Chung et al. as an empirical therapy for Proteus mirabilis. We also initiated the patient on a course of injection ceftriaxone along with vancomycin for a week until the arrival of culture-sensitivity report. The definitive therapy for Proteus mirabilis can vary geographically considering the drug resistance, prevalence and virulence of this bacterium. Hence, definitive therapy being an anti-bacterial targeting Gram-negative varied among documented cases.^6^,^7^

## CONCLUSION

Our case sums up the successful management of primary, multiple brain abscesses caused by Proteus mirabilis in a male child via surgical approach. Primary abscess caused by uncommon bacteria can present atypically. Hence, brain abscess should be considered as an important differential diagnosis in the scenario of multifocal space occupying lesions in brain, especially in immunocompetent pediatric population. Early surgical excision of the cyst is the mainstay of successful management.

### Authors Contribution

**TIK** supervised the project and critically reviewed the article.

**HMQ** conceived and designed the study, did literature search, wrote the manuscript draft and critically reviewed the article.

**IF and AG** contributed to literature review, data collection and manuscript writing.

**IF** responsible for the accuracy of the study.

**MTB** contributed to literature review and manuscript writing.

All authors have approved the final version of the manuscript.
